# The impact of health literacy on health-related quality of life among individuals with physical disabilities: a path analysis based on the integrated model of health literacy

**DOI:** 10.1007/s11136-026-04289-7

**Published:** 2026-06-06

**Authors:** Bohye Kim, Ju Young Yoon

**Affiliations:** 1 College of Nursing, Seoul National University, Seoul, South Korea; 2 Center for World-leading Human-care Nurse Leaders for the Future by Brain Korea 21 (BK 21) four project, College of Nursing, Seoul National University, Seoul, South Korea; 3 Research Institute of Nursing Science, Seoul National University, Seoul, South Korea

**Keywords:** Physical disabilities, Health-related quality of life, Health literacy, Unmet healthcare needs, Employment

## Abstract

**Purpose:**

This study examined the direct and indirect associations among health literacy (HL), its determinants, and health-related quality of life (HRQoL) in individuals with physical disabilities based on the Integrated Model of Health Literacy (IMHL).

**Methods:**

This cross-sectional study analyzed data from 441 adults with physical disabilities obtained from the 2021 Korea Health Panel Survey. Based on the IMHL, path analysis was conducted to examine the direct and indirect associations of situational determinants (usual source of care, need for care) and societal and environmental determinants (employment status, unmet healthcare needs) on HL and HRQoL.

**Results:**

Among the participants, 44.0% were classified as having inadequate HL and 22.3% as problematic. Need for care was negatively associated with both HL (β = − 0.12, *p* = .002) and HRQoL (β = − 0.33, *p* < .001). Unmet healthcare needs also had a significant negative direct association with HRQoL (β = − 0.17, *p* < .001). Employment status (β = 0.15, *p* < .001) and HL (β = 0.13, *p* = .005) were positively associated with HRQoL. HL partially mediated the relationship between need for care and HRQoL (β = − 0.02, *p* = .044).

**Conclusion:**

These findings highlight the role of HL in relation to HRQoL among individuals with physical disabilities. Improving HRQoL in this population may require approaches that go beyond individual-level HL promotion and also address support for care-dependent individuals and structural factors, including healthcare accessibility and employment opportunities.

## Abstract (plain language summary)

Individuals with physical disabilities often experience worse health and lower quality of life than the general population. This study examined whether health literacy affects health-related quality of life among individuals with physical disabilities in South Korea. It also explored how determinants of health literacy are linked to health literacy and health-related quality of life. The main finding is that health literacy plays an important role in health-related quality of life. The results showed that many participants had low health literacy. Those who needed more care or could not receive the healthcare they needed reported lower health-related quality of life. In contrast, individuals who had jobs and better health literacy reported better health-related quality of life. Health literacy helped explain why the need for care was linked to health-related quality of life. These findings suggest that helping individuals with physical disabilities better understand health information, making healthcare easier to access, and supporting employment opportunities may improve their health-related quality of life and reduce health inequalities.

## Introduction

Globally, health policy has shifted from disease treatment toward disease prevention and health promotion. Despite advances in medical technology and digital healthcare, health inequities remain a significant public health challenge [1]. In response, health literacy (HL) has gained increasing attention as a key strategy for reducing disparities in health resource utilization, and the World Health Organization (WHO) has emphasized HL as a core component of health promotion [2]. This is particularly important for vulnerable populations, who tend to have lower HL and experience greater health inequities [3].

Individuals with disabilities, a representative vulnerable population, tend to have poorer health status than those without disabilities and face multifaceted barriers such as economic burden and limited mobility, restricting access to healthcare [4]. Although they have a high need to seek and utilize health information independently, they often face difficulties with effective health management and decision-making due to low accessibility to health information [5]. Previous studies have consistently reported that individuals with disabilities demonstrate lower levels of HL, contributing to widening health disparities [5–8]. These disparities are further exacerbated during public health emergencies such as the COVID-19 pandemic [9].

Approximately 16% of the global population lives with a disability, a proportion rising due to increased life expectancy and growing non-communicable diseases [10]. In South Korea, physical disability — a condition in which upper and lower limb or body functions are limited due to paralysis, amputation, or deformity—accounts for approximately 45% of all registered persons with disabilities [11]. Individuals with physical disabilities show an increasing trend with age and have a high prevalence of chronic diseases [12, 13], and face constraints in healthcare utilization and low accessibility to health information [14–17]. Therefore, HL constitutes a critical factor in improving health equity and quality of life among this population [5].

HL refers to the ability to access, understand, appraise, and apply health-related information [18], and is recognized as a core determinant that directly influences health behaviors and health outcomes, including chronic disease management, preventive service use, and medication adherence, ultimately affecting quality of life [19, 20]. Within the Integrated Model of Health Literacy (IMHL), proposed by Sørensen et al. [18], HL is conceptualized as a mediating factor between antecedents and health outcomes. Among these outcomes, Health-related quality of life (HRQoL) reflects subjective health status and has been shown to be significantly associated with HL [21, 22].

Most previous research on HL has focused on the general population or individuals with chronic diseases [23], with limited attention given to people with physical disabilities [24]. A recent study among individuals with physical disabilities highlighted the role of digital HL in HRQoL [25]. However, few studies have examined the pathways through which general HL influences HRQoL in this population. Therefore, the present study aims to address this gap by applying the IMHL to identify both the direct and indirect pathways linking HL and HRQoL among individuals with physical disabilities in South Korea.

## Integrated model of health literacy (IMHL)

To address inconsistencies in existing HL definitions, Sørensen et al. [18] developed the IMHL, integrating clinical and public health perspectives. The IMHL delineates antecedent factors that influence HL, including societal and environmental determinants (e.g., demographic context, culture, societal system), personal determinants (e.g., gender, age, race, education), and situational determinants (e.g., social support, family and peer influences). These determinants affect HL, which in turn influences health outcomes and health costs.

The IMHL has been applied to diverse populations, including the general population [26–28], individuals with chronic conditions [29], patients [30], and immigrants [31]. These studies have consistently demonstrated that HL is significantly associated with various health outcomes. However, research applying the IMHL to individuals with disabilities remains limited. Therefore, the purpose of this study is to structurally examine the relationship between HL and HRQoL by applying the IMHL to individuals with physical disabilities. Based on the IMHL, we hypothesized that societal and environmental determinants, along with situational determinants, would be associated with HRQoL directly and indirectly through HL.

### Methods

#### Conceptual framework

**Fig. 1:**
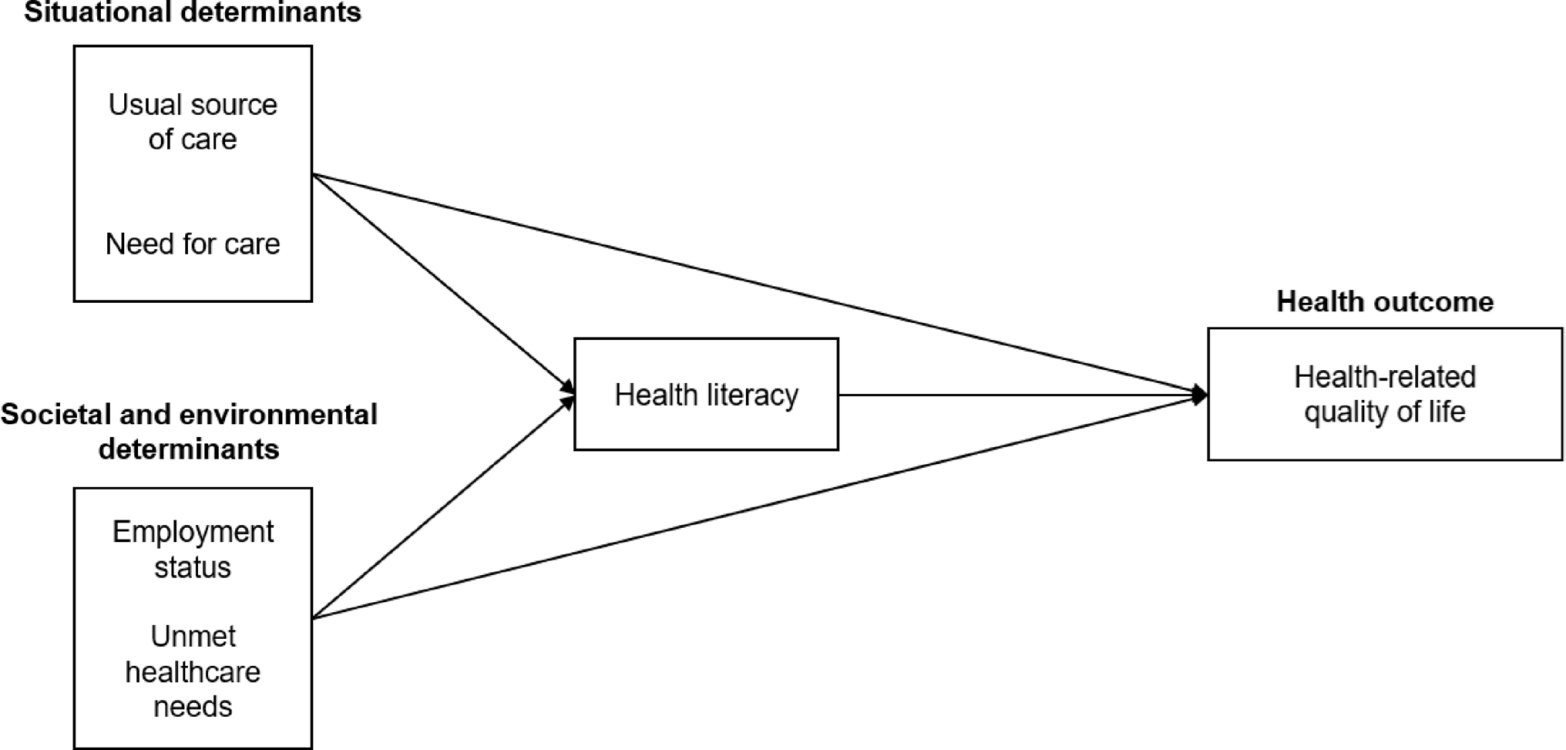
Conceptual framework of this study. Note. Personal characteristics (gender, age, education level, the number of chronic diseases, annual household income) were included as covariates in the analysis

Based on the IMHL, this study categorized four variables as antecedent determinants of HL. Usual source of care and need for care were classified as situational determinants, as they reflect proximal and immediate care circumstances [18]. Employment status was categorized as a societal and environmental determinant, following prior empirical validation [30], as it reflects structural barriers within the labor market faced by people with disabilities. Similarly, unmet healthcare needs were included as a societal and environmental determinant because disparities in healthcare utilization among people with disabilities are often shaped by structural and environmental barriers [32]. These variables have been consistently reported to be associated with both HL and HRQoL in previous studies [33–38]. Based on this framework, the analysis examined the effects of these determinants on the relationship between HL and HRQoL, controlling for personal determinants as covariates. Gender, age, the number of chronic diseases, and annual household income were controlled for both outcomes, whereas education was included only for HL due to its inconsistent association with HRQoL in vulnerable populations [39–45]. Therefore, the conceptual framework was designed to identify the direct and indirect pathways linking situational, societal and environmental determinants to HL and HRQoL among individuals with physical disabilities in South Korea (Fig. 1).

## Data source and study population

This study conducted a cross-sectional secondary analysis using the 2021 Korea Health Panel Survey (KHPS) data, version 2.2 [46]. The KHPS is a nationally representative panel survey conducted annually since 2008 by the Korea Institute for Health and Social Affairs and the National Health Insurance Service (NHIS). It provides fundamental data on healthcare utilization, medical expenditures, socioeconomic factors, health status and health behaviors of Korean household members. To ensure national representativeness, the KHPS adopted a two-stage stratified cluster sampling method, using the 2016 Population and Housing Census as the sampling frame. Since 2010, the survey has used the Computer-Assisted Personal Interviewing (CAPI) method. In the 2021 KHPS, a supplementary survey on HL was conducted among adult household members aged 19 years and older.

**Fig. 2:**
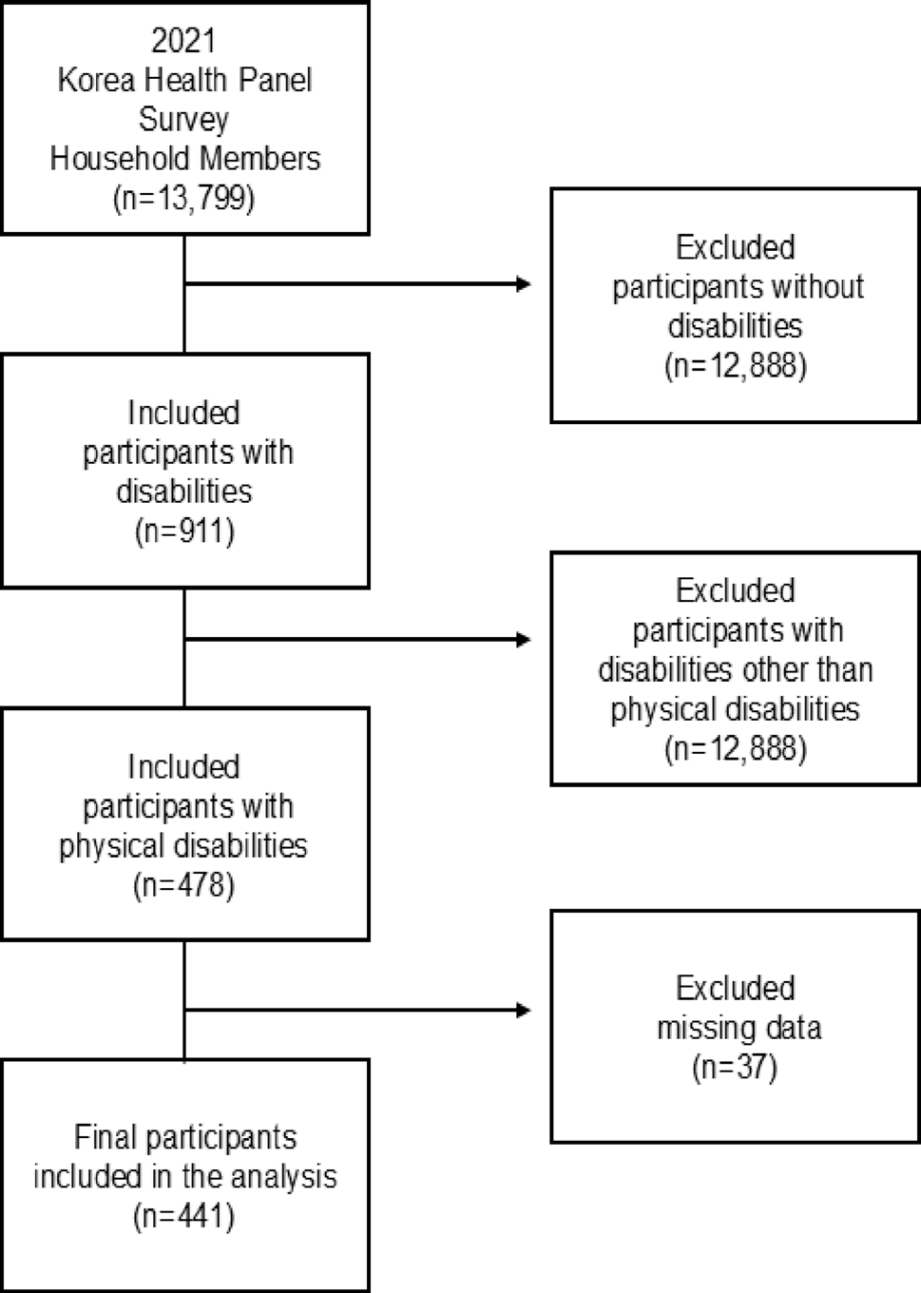
Flow chart of study population

The study population consisted of individuals with physical disabilities who participated in the 2021 KHPS. Of the 13,799 household members surveyed, 911 were identified as having disabilities, including 478 with physical disabilities. A total of 441 participants remained for the final analysis after excluding those with missing values for key variables, including HL and HRQoL (Fig. 2).

## Measurements

### Health-related quality of life (HRQoL)

HRQoL was measured using the Euro Quality of Life-5 Dimensions (EQ-5D). The EQ-5D assesses five dimensions of HRQoL including mobility, self-care, usual activities, pain/discomfort, and anxiety or depression, each with three levels (no problems, some problems, and extreme problems). The Korean version of the EQ-5D has been validated and shown to have acceptable reliability and validity [47]. To calculate the index scores, we applied Korean-specific value sets developed to reflect the health preferences of the Korean population [48] The index scores range from − 0.171 to 1, with higher scores reflecting better health.

### Health literacy (HL)

HL was assessed using the European Health Literacy Survey Q16 (HLS-EU-Q16) from the HL survey conducted in 2021 as a supplement to the KHPS [49]. The HLS-EU-Q16 is a shortened version of the 47-item HLS-EU-Q47, comprising 16 items categorized into three domains: health care (7 items), disease prevention (5 items), and health promotion (4 items). It was assessed across four cognitive dimensions: accessing, understanding, appraising, and applying health information [50]. This tool has been widely used across diverse populations to validate its applicability in different cultural contexts [51]. This study employed the Korean version of the HLS-EU-Q16, which was translated and adapted by Cheon and Lee [52]. In scoring the HLS-EU-Q16 items assessing HL, responses of “very difficult” or “difficult” were coded as 0, and responses of “easy” or “very easy” were coded as 1 [49]. The scores were then summed to produce a total HL score ranging from 0 to 16, with higher scores indicating higher HL levels. HL levels were classified as inadequate (0–8), problematic (9–12), or adequate (13–16) [53, 54].

## Determinants of HL

The presence of a usual source of care and need for care were used to assess situational determinant of HL. The presence of a usual source of care was dichotomized (yes/no) based on whether the individual reported having a regular medical institution or physician they typically visit when sick or in need of consultation. The need for care was dichotomized (yes/no) according to whether the individual experienced difficulties in daily, social, or leisure activities due to illness or injury, thereby requiring supportive care.

Employment status and unmet healthcare need were included as societal and environmental determinants of HL. Employment status was categorized as employed or unemployed. Unmet healthcare needs were dichotomized (yes/no) based on whether individuals reported any difficulty accessing medical care when needed.

Personal determinants of HL included as covariates were gender, age, education level, the number of chronic diseases, and annual household income. Gender was dichotomized into male or female. Age was divided into four groups: 39 years or younger, 40–59 years, 60–79 years, and 80 years or older. Education level was classified into four categories: elementary school or below, middle school, high school, and college or above. The number of chronic diseases was defined as the total count of chronic conditions diagnosed by a physician. Annual household income was categorized into quartiles: low (≤ 17.79 million Korean won, KRW), low-middle (17.80–35.88 million KRW), high-middle (35.89–61.24 million KRW), and high (≥ 61.25 million KRW).

### Statistical analysis

The KHPS employed a probability-proportional, two-stage, stratified cluster sampling method to provide nationally representative estimates. In this study, the complex survey design and sampling weights were applied to ensure that the sample reflected the target population. Participants with missing values for key variables were excluded, resulting in a final analytical sample of 441 individuals. In complex survey analysis, to prevent standard error bias that can occur when cases are deleted or selectively analyzed, a grouping variable was created and used to define the parent population for analysis [55]. Descriptive statistics, including weighted percentages, means, and standard errors, were calculated using the complex samples module in SPSS to examine the research variables. Differences in HL and HRQoL according to participant characteristics were assessed using the complex samples general linear model with Bonferroni correction. All complex samples analyses were performed using SPSS version 29.0, and a *p-*value of < 0.05 was considered statistically significant.

The research model was assessed through path analysis using Mplus version 8.9. For the path analysis, unweighted data were used to prioritize model stability, as applying complex sampling weights within the path model risked producing unstable parameter estimates or convergence issues given the relatively small size of certain subgroups (e.g., need for care, *n* = 53). To verify the assumptions of path analysis, multicollinearity among independent variables was assessed using tolerance and variance inflation factor (VIF) values [56]. Model parameters were estimated using the maximum likelihood method. Model fit was evaluated based on four indices, including chi-square (χ2) statistics, comparative fit index (CFI), Tucker–Lewis index (TLI), standardized root mean square residual (SRMR), and root mean square error of approximation (RMSEA). CFI and TLI values of 0.95 or higher indicate acceptable to good model fit, with higher values reflecting better fit [56]. SRMR values closer to 0 indicate better model fit, with values below 0.05 considered acceptable [56]. RMSEA values of 0.05 or less indicate good model fit, whereas values between 0.06 and 0.08 suggest acceptable model fit [56]. Direct, indirect, and total effects were examined using bootstrapping with 5,000 resamples and 95% confidence intervals (CI).

### Ethical considerations

This study was a secondary analysis of de-identified, publicly available data from the KHPS. Ethical approval was obtained from the Institutional Review Board of Seoul National University (IRB No. E2403/002–012), with a waiver of informed consent granted due to the use of de-identified retrospective data. All procedures were conducted in accordance with relevant ethical guidelines and regulations.

## Results

Table 1 presents descriptive statistics of the participants’ determinants of HL, HL, and HRQoL. Overall, 68.9% had a usual source of care, 13.2% were in need of care, 42.9% were employed, and 23.1% reported unmet healthcare needs. The mean HL score was 9.45 ± 0.3, with 44.0% classified as having inadequate HL, 22.3% as problematic, and 33.7% as adequate. The mean HRQoL score was 0.84 ± 0.01. As shown in Table 2, HL differed significantly by gender, age group, and education level. Males had significantly higher HL scores than females (t = -2.52, *p* = .012). Significant differences were observed across age groups (F = 11.91, *p* < .001), with post-hoc Bonferroni tests indicating that participants aged ≤ 39 years scored higher than those in the 40–59, 60–79, and ≥ 80 age groups (*p* < .05), and that the 40–59 and 60–79 age groups scored higher than the ≥ 80 group (*p* < .05). HL also differed by education level (F = 13.24, *p* < .001). Post-hoc Bonferroni tests showed that participants with an elementary school education or below had significantly lower scores than those with a middle school, high school, or college education (*p* < .05). Additionally, the middle school group scored significantly lower than the college group (*p* < .05). There were statistically significant differences in HL according to chronic disease (t = 3.24, *p* < .001), need for care (t = 2.29, *p* = .022), and employment status (t = -2.20, *p* = .028).

**Table 1:** Descriptive statistics of participants’ determinants of health literacy, health literacy, and HRQoL (*n* = 441)

Variables		Categories (Range)	*n* (%) or M ± SE
Personal determinants	Gender	Male	219 (54.2)
		Female	222 (45.8)
	Age	≤ 39	4 (2.2)
		40–59	59 (25.9)
		60–79	296 (55.5)
		≥ 80	82 (16.4)
	Education	≤ Elementary school	234 (42.8)
		Middle school	80 (17.3)
		High school	84 (24.4)
		≥ College	43 (15.5)
	Annual household income	Low	180 (37.5)
		Low middle	136 (25.4)
		High middle	77 (22.2)
		High	48 (15.0)
	Chronic disease	Yes	403 (83.8)
		No	38 (16.2)
		The number of chronic diseases (0–12)	2.45 ± 0.12
Situational determinants	Usual source of care	Yes	339 (68.9)
		No	102 (31.1)
	Need for care	Yes	53 (13.2)
		No	388 (86.8)
Societal and environmental determinants	Employment status	Yes	189 (42.9)
		No	252 (57.1)
	Unmet healthcare needs	Yes	86 (23.1)
		No	355 (76.9)
Health literacy	Health literacy	Inadequate	230 (44.0)
		Problematic	101 (22.3)
		Adequate	110 (33.7)
		Total (0–16)	9.45 ± 0.31
Health outcome	HRQoL	(-0.17–1)	0.84 ± 0.01

Unweighted n (weighted %); M = Estimated mean; SE = Standard Error; HRQoL = Health related quality of life

**Table 2:** Differences in health literacy according to determinants of health literacy (*n* = 441)

Variables		Categories (Range)	M ± SE	t of F	*p* (post hoc)
Personal determinants	Gender	Male	10.80 ± 0.48	− 2.52	0.012
		Female	9.41 ± 0.61		
	Age	≤ 39^a^	13.54 ± 0.53	11.91	< 0.001 (a > b,c, d; b, c > d)
		40-59^b^	11.59 ± 0.59		
		60-79^c^	10.13 ± 0.32		
		≥ 80^d^	7.98 ± 0.68		
	Education	≤ Elementary school^a^	8.34 ± 0.46	13.24	< 0.001 (a < b,c, d; b < d)
		Middle school^b^	10.19 ± 0.63		
		High school^c^	11.82 ± 0.50		
		≥ College^d^	12.90 ± 0.47		
	Annual household income	Low	9.82 ± 0.43	2.45	0.062
		Low middle	11.14 ± 0.45		
		High middle	10.90 ± 0.53		
		High	11.38 ± 0.50		
	Chronic disease	Yes	8.50 ± 0.41	3.24	0.001
		No	11.71 ± 0.88		
Situational determinants	Usual source of care	Yes	10.10 ± 0.60	0.03	0.979
		No	10.12 ± 0.57		
	Need for care	Yes	9.35 ± 0.66	2.29	0.022
		No	10.87 ± 0.48		
Societal and environmental determinants	Employment status	Yes	10.76 ± 0.56	− 2.20	0.028
		No	9.45 ± 0.55		
	Unmet healthcare needs	Yes	10.27 ± 0.62	− 0.58	0.566
		No	9.94 ± 0.48		

M = Estimated mean; SE = Standard error

Differences in HRQoL are presented in Table 3. Although HRQoL differed significantly across education levels (F = 3.10, *p* = .026), post-hoc Bonferroni tests showed no significant pairwise differences. Participants with need for care reported lower HRQoL than those without (t = 3.71, *p* < .001). HRQoL was higher among employed participants compared to those who were not employed (t = − 3.57, *p* < .001), and lower among participants with unmet healthcare needs compared to those without (t = 3.14, *p* = .002).

**Table 3:** Differences in HRQoL according to determinants of health literacy (*n* = 441)

Variables		Categories (Range)	M ± SE	t of F	*p* (post hoc)
Personal determinants	Gender	Male	0.76 ± 0.04	− 0.25	0.803
		Female	0.76 ± 0.04		
	Age	≤ 39^a^	0.74 ± 0.12	1.12	0.342
		40-59^b^	0.84 ± 0.03		
		60-79^c^	0.87 ± 0.01		
		≥ 80^d^	0.84 ± 0.03		
	Education	≤ Elementary school	0.78 ± 0.04	3.10	0.026 (NS)
		Middle school	0.82 ± 0.04		
		High school	0.84 ± 0.05		
		≥ College	0.85 ± 0.03		
	Annual household income	Low	0.78 ± 0.04	1.84	0.138
		Low middle	0.84 ± 0.03		
		High middle	0.82 ± 0.04		
		High	0.85 ± 0.03		
	Chronic disease	Yes	0.76 ± 0.02	− 0.03	0.973
		No	0.76 ± 0.05		
Situational determinants	Usual source of care	Yes	0.74 ± 0.04	1.76	0.079
		No	0.78 ± 0.04		
	Need for care	Yes	0.67 ± 0.06	3.71	< 0.001
		No	0.85 ± 0.02		
Societal and environmental determinants	Employment status	Yes	0.79 ± 0.04	− 3.57	< 0.001
		No	0.73 ± 0.04		
	Unmet healthcare needs	Yes	0.72 ± 0.04	3.14	0.002
		No	0.80 ± 0.03		

M = Estimated mean; SE = Standard error; HRQoL = Health related quality of life; NS = Not significant

Prior to conducting the path analysis, no multicollinearity was detected. Tolerance values ranged from 0.21 to 0.93, and VIF values ranged from 1.08 to 4.84. The path model demonstrated a good model fit (χ^2^ = 4.307, *p* = .230, CFI = 0.996, TLI = 0.957, RMSEA = 0.031, SRMR = 0.013).

**Fig. 3:**
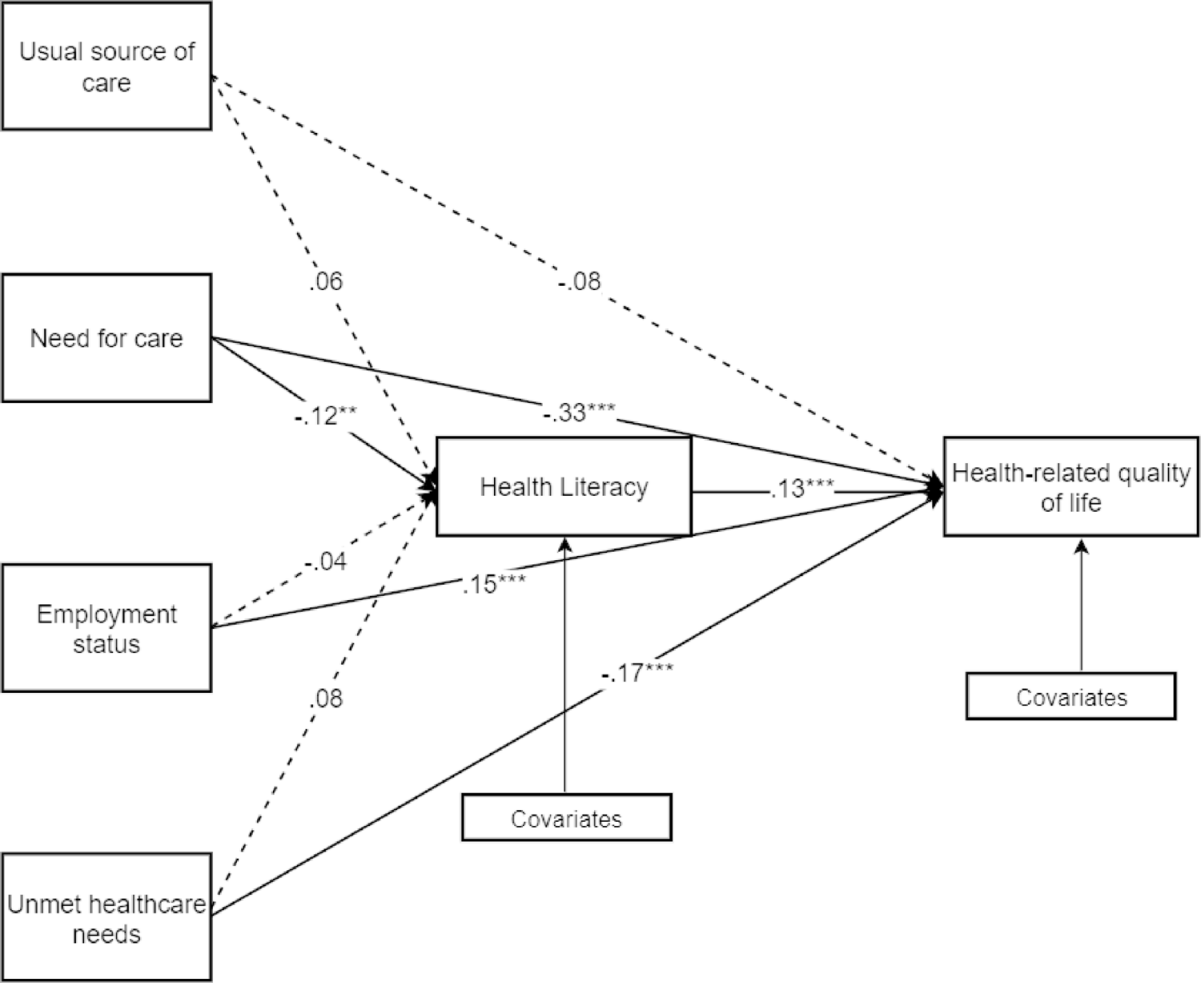
Path estimates of the model. Note. Solid lines show significant paths and dotted lines are not statistically significant paths. All coefficients are standardized. Covariates for health literacy (gender, age, education level, the number of chronic disease and annual household income) and Health-related quality of life (gender, age, the number of chronic disease and annual household income) were included in the path model. *p < .05, **p < .01, ***p < .001

Table 4; Fig. 3 present the significance of the model. Need for care (β = − 0.12, *p* = .002) exhibited a significant and negative relationship with HL. For HRQoL, need for care (β = − 0.33, *p* < .001), and unmet healthcare needs (β = − 0.17, *p* < .001) demonstrated a negative direct relationship. Employment status was positively associated with HRQoL (β = 0.15, *p* < .001), while HL was also positively associated with HRQoL (β = 0.13, *p* = .005). 

Table 5 presents the mediating role of HL. HL showed significant partial mediation (β = −0.02, p = .044) in the relationship between need for care and HRQoL. In contrast, HL did not show significant mediation in the relationships among usual source of care (β = 0.01, p = .221), employment status (β = −0.01, p = .400), and unmet healthcare needs (β = 0.01, p = .143) with HRQoL.

**Table 4:** Results of path analysis

Model pathway		*β*	S.E	p	95% CI	R^2^
Health literacy	← Usual source of care	0.06	0.04	0.154	− 0.290, 1.663	0.356
	← Need for care	**− 0.12**	**0.04**	**0.002**	**− 2.921**,** − 0.736**	
	← Employment status	− 0.04	0.04	0.332	− 1.235, 0.419	
	← Unmet healthcare need	0.08	0.04	0.050	− 0.018, 1.865	
HRQoL	← Usual source of care	− 0.08	0.04	0.060	− 0.064, 0.000	0.279
	← Need for care	**− 0.33**	**0.06**	**< 0.001**	**− 0.231**,** − 0.095**	
	← Employment status	**0.15**	**0.04**	**< 0.001**	**0.022**,** 0.072**	
	← Unmet healthcare need	**− 0.17**	**0.04**	**< 0.001**	**− 0.100**,** − 0.035**	
	← Health literacy	**0.13**	**0.05**	**0.005**	**0.001**,** 0.007**	

Values are standardized coefficients; S.E = Standard error; CI = Confidence intervals; HRQoL=Health-related quality of life; Bold values indicate significant results

**Table 5:** The mediation role of health literacy

Model pathway		Effect	β	S.E	*p*	95% CI
HRQoL	← Usual source of care	Total	− 0.07	0.04	0.088	− 0.061, 0.003
		Indirect	0.01	0.01	0.221	− 0.001, 0.009
	← Need for care	**Total**	**− 0.34**	**0.06**	**< 0.001**	**− 0.238**,** − 0.103**
		**Indirect**	**− 0.02**	**0.01**	**0.044**	**− 0.016**,** − 0.002**
	← Employment status	**Total**	**0.14**	**0.04**	**0.001**	**0.020**,** 0.071**
		Indirect	− 0.01	0.01	0.400	− 0.007, 0.001
	← Unmet healthcare need	**Total**	**− 0.16**	**0.04**	**< 0.001**	**− 0.098**,** − 0.031**
		Indirect	0.01	0.01	0.143	0.000, 0.011

Values are standardized coefficients; S.E = Standard error; CI = Confidence intervals; HRQoL = Health-related quality of life; Bold values indicate significant results

## Discussion

This study examined the direct and indirect pathways linking HL and HRQoL among individuals with physical disabilities in South Korea based on the IMHL. The findings indicated that HL showed a significant direct association with HRQoL and partially mediated the relationship between need for care and HRQoL. In addition, need for care was associated with lower HL and HRQoL, while unmet healthcare needs and employment status were also associated with HRQoL.

The results showed that the overall level of HL among individuals with physical disabilities was relatively low, with more than half of the participants classified as having inadequate or problematic HL. This finding is consistent with previous studies reporting low HL levels among individuals with disabilities [5, 8], and suggests that individuals with physical disabilities face substantial barriers to effective use of health information. In particular, for individuals with physical disabilities, ease of use becomes a greater consideration than the usefulness of information in the process of seeking and utilizing health information due to physical constraints, and this becomes more pronounced as the degree of disability increases [57]. This finding suggests that accessibility demands related to physical functioning may influence how this population searches for and use health information, leading to reliance on information that is easier to access and navigate [57]. However, lower HL should not be interpreted solely as an individual limitation. From a biopsychosocial perspective reflected in the WHO’s International Classification of Functioning, Disability and Health (ICF), HL arises from the interaction between personal characteristics and environmental and social conditions [58]. From this perspective, individuals with physical disabilities may encounter systemic and environmental barriers within healthcare systems, including communication barriers, disability stigma, and reliance on caregivers for information exchange, which may limit opportunities to access and utilize health information [59]. Therefore, improving HL in this population requires not only strengthening individual competencies but also reducing environmental and systemic barriers to accessible health information.

In this study, need for care, a situational determinant, showed a negative association with HL among individuals with physical disabilities. This finding shows a similar trend to a previous study that identified lower HL among older adults with chronic conditions who have high levels of functional dependence [60]. Individuals with physical disabilities often rely on caregivers during routine health management and healthcare utilization. This care-dependent environment may reduce their ability to actively seek and understand health information and participate autonomously in medical decision-making processes [17]. Particularly when communication between caregivers and healthcare providers bypasses the individual or fails to reflect their level of understanding and information needs, individuals with physical disabilities experience difficulties in directly acquiring needed health information. In healthcare settings where autonomy is not sufficiently ensured, opportunities for improving HL may be constrained [61]. Therefore, those in care-dependent situations may represent a vulnerable group, highlighting the need for targeted support.

In addition to its association with HL, need for care showed a direct negative association with HRQoL, highlighting care dependency as an important situational factor in shaping quality of life in individuals with physical disabilities. The findings also indicate that HL partially mediated the relationship between need for care and HRQoL, suggesting an indirect pathway through which care-dependent contexts may influence health outcomes. Care dependence may limit individuals’ opportunities to access, understand, and apply health information [17], which may in turn affect their ability to participate in treatment-related decision-making, engage in self-management, and effectively navigate healthcare services [62, 63], ultimately contributing to poorer HRQoL. These findings support the IMHL by empirically illustrating how situational determinants are linked to health outcomes through HL in this population [18]. Therefore, to improve HRQoL among those in care-dependent situations, interventions should address not only individual-level HL support but also caregiving and healthcare communication contexts that influence autonomy and participation in health-related decision-making, including inclusive communication and shared decision-making with caregiver involvement [64]. Future research may examine how different caregiving arrangements and healthcare interaction contexts influence HL development and subsequent health outcomes.

Regarding societal and environmental determinants, unmet healthcare needs were significantly associated with poorer HRQoL among individuals with physical disabilities. This finding is consistent with previous studies suggesting that unmet healthcare needs are negatively associated with quality of life regardless of disability status [37, 65, 66]. Prior research has reported higher levels of unmet healthcare needs among this population compared to the general population and other disability groups, largely due to barriers related to accessibility and transportation [67]. This finding suggests that mobility barriers and structural limitations in healthcare access may contribute to unmet healthcare needs, potentially creating a vicious cycle that exacerbates health deterioration and reduces quality of life. Therefore, improving HRQoL in this population may require efforts that go beyond the quantitative expansion of healthcare services. Organizations and healthcare providers need to focus on creating disability-friendly healthcare environments, strengthening mobility and transportation support, and improving healthcare accessibility.

Employment status was also significantly associated with HRQoL, with employed individuals with physical disabilities reporting higher HRQoL compared to their unemployed counterparts. This finding aligns with previous research demonstrating the positive association between employment status and HRQoL among people with disabilities [68–70]. Employment provides not only economic benefits but also opportunities for social participation and self-realization, thereby positively contributing to psychological and social well-being [71–73]. Furthermore, the type and conditions of employment may influence well-being, with more supportive employment associated with better quality of life [74]. Despite its significant role in shaping health and well-being, employment rates in this population remain substantially lower than those of the general population [75]. They continue to face multiple barriers, including the need for assistive technologies and workplace accommodations, discriminatory attitudes toward disability, and limited accessibility related to transportation and mobility [76]. Consequently, improving HRQoL in this population may require multifaceted efforts to enhance employment accessibility, including strengthening employment promotion policies, creating accessible work environments, and expanding vocational training and rehabilitation programs.

However, HL did not mediate the relationships between these societal and environmental determinants and HRQoL. Employment status and unmet healthcare needs showed significant direct associations with HRQoL but were not linked through HL, suggesting that these may operate more directly through structural conditions in relation to HRQoL [77]. In addition, usual source of care was not significantly associated with either HL or HRQoL, which may reflect the limitation of the measure, as it captures only the presence of a regular provider and not the quality or nature of healthcare interactions. Future research may benefit from incorporating measures of care quality and patient–provider interactions.

This study has several limitations. First, the cross-sectional design limits the ability to establish causal relationships among variables. Future longitudinal studies are needed to better capture dynamic relationships over time. Second, the use of secondary data constrained the range of available variables. Important disability-related factors, such as disability severity and duration, were not included, which may contribute to residual confounding and limit interpretation of the findings. Future research should incorporate these variables to better understand HL and HRQoL in this population. Third, although validated instruments were used, HL and HRQoL measures may not fully capture disability-specific contexts, which may limit interpretation of the findings. Fourth, as the study sample was limited to individuals with physical disabilities, caution is warranted in generalizing the findings to other disability types. Thus, future comparative studies across different disability groups are recommended to examine whether the relationships between HL and HRQoL differ by disability type. Fifth, certain subgroups were relatively small in size, which may limit the stability of the estimated path coefficients. Accordingly, these results should be interpreted with caution. Sixth, although standardized path coefficients were reported as indicators of effect size, the observed coefficients ranged from relatively small to moderate, and the practical significance of these findings should be interpreted with caution. Future studies with larger and more diverse samples are needed to further validate these structural relationships.

Despite these limitations, this study contributes to the IMHL framework by applying it to individuals with physical disabilities and identifying HL as a contextually embedded pathway linking care dependence to HRQoL. These findings suggest that improving HRQoL in this population requires approaches beyond individual-level HL. Interventions may benefit from integrating HL promotion strategies for care-dependent individuals with efforts to address structural barriers, including healthcare access and employment.

## Conclusion

This study empirically examined the associations of HL and its determinants with HRQoL and the mediating pathways involving HL among individuals with physical disabilities based on the IMHL. The results showed that HL had a significant direct association with HRQoL and a partial mediating role in the association between need for care and HRQoL. Additionally, unmet healthcare needs and employment status were identified as significant factors associated with HRQoL. These findings suggest that improving HRQoL for individuals with physical disabilities may require an integrated, multidimensional approach that goes beyond individual-level HL promotion by strengthening support for care-dependent individuals, improving healthcare accessibility, and expanding employment opportunities.

## Data Availability

The datasets used in this study are from the Korea Health Panel Survey and are not publicly available due to access restrictions, but are available through an application process at its official website (https://www.khp.re.kr).
